# Autism and self‐harm: A population‐based and discordant sibling study of young individuals

**DOI:** 10.1111/acps.13479

**Published:** 2022-08-03

**Authors:** Isidora Stark, Dheeraj Rai, Michael Lundberg, Iryna Culpin, Selma Idring Nordström, Anna Ohlis, Cecilia Magnusson

**Affiliations:** ^1^ Karolinska Institutet Department of Global Public Health Stockholm Sweden; ^2^ Centre for Academic Mental Health, Population Health Sciences Bristol Medical School Bristol UK; ^3^ Department of Psychology Manchester Metropolitan University Manchester UK; ^4^ Centre for Psychiatry Research Department of Clinical Neuroscience Stockholm Sweden

**Keywords:** autism, mental health, population‐based cohort, self‐harm, self‐injurious behavior

## Abstract

**Objective:**

Self‐harm among young autistic individuals is a clinical challenge, and the risk of premature death by suicide is strongly increased in this group. Using the advantage of total‐population and family‐based data, we investigated whether autism per se is a risk factor for self‐harm independently of psychiatric comorbidities and how it differs from self‐harm in non‐autistic individuals.

**Methods:**

We used The Stockholm Youth Cohort, a total‐population register study, including all residents in Stockholm County aged 0–17 years between 2001 and 2011.Study participants were followed from age 10 to 27 for hospital admissions because of self‐harm. We used modified Poisson regression to calculate relative risks (RR) using robust standard error to derive 95% confidence intervals (CI).

**Results:**

In all, 410,732 individuals were included in the cohort (9,070 with a diagnosis of autism). Autistic individuals had a fivefold increased adjusted relative risk of self‐harm (RR 5.0 [95% CI 4.4–5.6]). The risk increase was more pronounced for autism without intellectual disability and particularly high for self‐cutting 10.2 [7.1–14.7] and more violent methods 8.9 [5.2–15.4]. The association between autism and self‐harm was independent of, but clearly exacerbated by comorbid psychiatric conditions. It was of similar magnitude as risks linked to these conditions per se, and not explained by shared familial factors.

**Conclusion:**

Self‐harm severe enough to present to medical services is as common in autistic youth as in those with depression or ADHD. Potentially more lethal methods are more likely to be used of autistic self‐harmers.

**Significant Outcomes**

- Autism should be regarded as a risk‐factor for self‐harm and addressed in clinical guidelines for suicide prevention even if not presented with depression, anxiety disorder, and ADHD methods of self‐harm in autistic individuals, particularly those without intellectual disability, may be more lethal and should be addressed in future studies.

**Limitations**

- The limitation of this study is that we investigated self‐harm registered by medical services, therefore results may not apply to self‐harm that occur without presentation to services.

## 1 INTRODUCTION

Self‐harm is the second leading cause of death and disability in young individuals globally^1^, ^2^ and a major public health concern. It is a complex and multi‐determined behavior^3^ and refers to intentional self‐poisoning or self‐injury, regardless of the motive or suicidal intent. It includes a continuum of behaviors from non‐suicidal self‐injury to completed suicide.^4^ Self‐harm is a strong predictor of completed suicide,^5^, ^6^, ^7^ and if not fatal associated to long‐term problems including increased risk of ill health,^8^, ^9^ substance misuse,^10^ and a range of socioeconomic disadvantages.^11^, ^12^, ^13^, ^14^

Autism is a broad diagnostic category encompassing developmental conditions characterized by difficulties with reciprocal social interaction and restricted and repetitive patterns of interests and behaviors^15^ presenting either with or without intellectual disability (ID). Autistic individuals are at high risk of mental‐health problems and suicidality.^16^, ^17^ Although increasing numbers of individuals are being diagnosed with autism,^18^, ^19^ there is still limited understanding of unique healthcare needs of these individuals.^17^, ^20^ It has been hypothesized that autism itself should be considered a risk factor for suicidal behaviors^17^, ^21^ This is because autistic individuals are exposed to risk factors for such behaviors, including social isolation, low self‐esteem, or feelings of social exclusion, and often have comorbid psychiatric conditions known to be strongly linked to self‐harm in general population^3^, ^4^, ^22^, ^23^ and increased risks of suicidal behaviors (including self‐harm) have been noted in autistic individuals.^24^, ^25^ Yet, more robust empirical evidence for this notion has been called for and autism is yet not recognized in recent clinical guidelines for suicide prevention.^26^

Findings regarding the influence of psychiatric comorbid conditions on self‐harm in autism are few and their results conflicting.^22^, ^23^, ^27^ In a study from Finland, the increased risk of intentional self‐harm in young autistic individuals was explained by comorbid psychiatric conditions.^22^ In contrast, a cohort study from Taiwan showed that autism was an independent risk factor for self‐harm.^23^ ADHD, depression, and anxiety disorder are common in autism^16^, ^28^ and are well‐established risk factors for self‐harm in general population.^28^, ^29^ With the regard to clinical utility of risk‐factors in assessment of self‐harming individuals, there is a need to determine whether autism per se increases risk of self‐harm independently of these conditions. Moreover, more precise estimates of the risk of self‐harm in autism, and whether it is comparable with those of ADHD, depression, and anxiety disorders solely, may inform clinical decision making.

Hirvikovski and colleagues have reported a marked increased risk of suicide in autism^25^ particularly in autistic individuals without ID,^25^ who may have greater cognitive abilities to conceptualize, plan and engage in suicidal behaviors.^30^, ^31^ Self‐harm is a possibly intervenable precursor of suicide, and knowledge on whether and how it differs in autism according to comorbid ID is needed to inform adjustments in preventive actions to meet unique needs in these two subgroups.

Suicidal behavior is known to aggregate in families,^4^ because of shared genetic as well as environmental factors. There is also evidence of a moderate genetic correlation between autism and intentional self‐harm.^32^ Family‐based studies may shed light on familial risks and help disentangle effects of shared environmental and genetic factors. A Swedish study found a higher risk of suicide attempts and death by suicide in full siblings than in half‐siblings of autistic individuals, thus indicating that genetic rather than environmental factors explain familial associations between autism and self‐harm.^27^ However, this study needs confirmation, particularly in a more recent cohorts where autism has been more broadly diagnosed.

While self‐harm is a risk factor for completed suicide overall, the level of risk increase varies considerably between methods of self‐harm in general population.^33^ For example, more violent self‐harm methods (e.g., hanging, drowning, use of firearms or explosives, jumping from a height, or gassing) confer a much poorer prognosis than self‐harm by poisoning or cutting.^28^ Yet, there is some data that self‐harm methods differ between autistic and non‐autistic individuals,^24^ and self‐cutting has been found to be relatively less common in the autistic population^17^ than in the general community.^34^ Yet, large‐scale studies on this topic are missing.

### 1.1 Aims of this study

- To clarify whether autism increases risk for self‐harm independent of comorbid depression, anxiety disorders, and ADHD, and how this risk‐increase compares to risks of self‐harm because of depression, anxiety disorders, and ADHD;
- To investigate whether risks of self‐harm vary between individuals with autism according to co‐existing intellectual disability;
- To investigate if family level confounding can explain any link between autism and self‐harm;
- To clarify whether self‐harm methods differ between autistic and non‐autistic individuals.

## 2 MATERIAL AND METHODS

### 2.1 Study population and design

**FIGURE 1:**
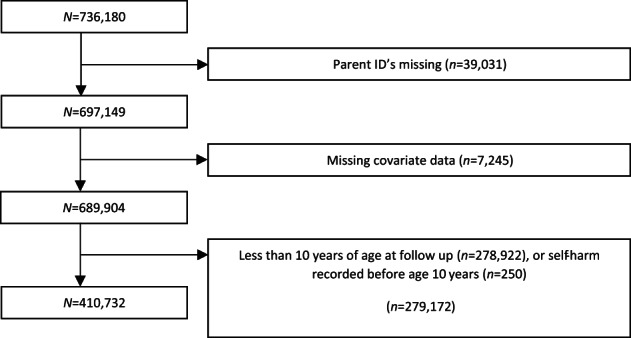
Derivation of the analytic sample

The Stockholm Youth Cohort (SYC) is a total population record‐linkage study which includes all children and young people, up to 17 years old, who were ever resident in Stockholm County, Sweden between 2001 through 2011 (total *N* = 735,096).^35^ The SYC comprises data on cohort members and their first and second‐degree relatives, collected using a range of Swedish national and regional registries. The cohort was followed‐up until December 31, 2011, when the oldest study participants were 27 years of age. Our primary outcome was a record of admission for self‐harm, and the analytical sample was restricted to individuals who were at least 10 years of age (*n* = 410,732, Figure 1) in 2011 to exclude accidental self‐harm characteristic of small children.

We identified the full (those who had the same biological parents as the index person) and half siblings (those with one common biological parent) of these individuals using the Swedish Multigenerational register.^36^

### 2.2 Ascertainment of autism

Autism was identified using record linkage with registries covering all known publicly funded pathways of assessment, care, or special education for autism in Stockholm County.^35^ Using this multisource ascertainment method, 12,259 (1.67%) individuals within the cohort were classified as having autism if been diagnosed according to the World Health Organization's International Classification of Diseases (ICD) by codes ICD‐9‐299, ICD‐10 F84, and Diagnostic and Statistical Manual of Mental Disorders (DSM‐IV) 299 and by the denotation of autism by habilitation services^37^ during the study period. We have previously carried out two validation studies including a case note review by clinical experts, and a cross validation study with a national study of twins, which both highlighted the validity of the cases recorded as having autism in the SYC.^35^

Apart from studying autism as a combined group, (including ICD‐10 codes F84.0 autistic disorder, F84.1 atypical autism, F84.5 Asperger syndrome, and F84.9 pervasive developmental disorder‐not otherwise specified) we also dichotomized autism based on the presence of recorded ID in the registers.^35^ Diagnosis of ID was identified in accordance with codes: ICD‐9: 319, ICD‐10: F70–F79, DSM‐IV 317–319.

### 2.3 Ascertainment of self‐harm

We used the Swedish National Patient Register to identify individuals within the SYC who had hospital admissions (including emergency department visits) with a discharge diagnosis of self‐harm, as defined by the, ICD‐10 codes X60‐X84 (purposely self‐inflicted poisoning or injury) and Y10‐Y34 (events of undetermined intent). We used any self‐harm irrespective of a recorded intent for our main analysis.

We also categorized self‐harm according to method, i.e. as poisoning (ICD‐9: 950‐952; 980‐982; ICD‐10 X60‐X69, Y10‐Y19), cutting (ICD‐9: 956, 986; ICD‐10 X78, Y28 ***)***, and violent forms of self‐harm “violent methods” (hanging /strangulation, drowning, firearm, jumping from a high place, jumping or lying before moving object; ICD‐9: 953‐957, 983‐987; ICD 10: X70‐X75, X80,X81,Y20‐25, Y30, Y31), with or without known intent, respectively.

### 2.4 Ascertainment of covariates

We included the following variables as potential confounders: age and sex of the index person, birth order (first, second, third, or greater), paternal and maternal age at birth,^38^ highest educational qualification of either parent (≤9 years, 10–12, ≥13 years) and disposable household income adjusted for year of ascertainment and family size (in quintiles) closest to birth,^39^ migrant household, defined as a parent or the index person born outside of Sweden,^40^ and any history of a psychiatric disorder in the mother or the father.^41^

We used the National patient register, the Stockholm Adult Psychiatry register and the Stockholm County child and adolescent mental health services register (The Pastill Register) to identify clinically recorded diagnoses of attention deficit hyperactivity disorder (ADHD) identified by ICD‐10 codes (F90.0, Attention‐deficit hyperactivity disorder, predominantly inattentive type; F90.1, Attention‐deficit hyperactivity disorder, predominantly hyperactive type; F90.2, Attention‐deficit hyperactivity disorder, combined type; F90.8, Attention‐deficit hyperactivity disorder, other type; F90.9, Attention‐deficit hyperactivity disorder, unspecified type).

We identified clinically recorded diagnoses of depressive disorders (ICD‐10 F32‐39), anxiety and stress related disorders (ICD‐10 F40‐48) in index persons. To minimize any possibility of reverse causality leading to spurious results (e.g., depressive disorders being diagnosed because of admission for self‐harm), we required the diagnosis or depression or anxiety disorders to occur before the date of recorded self‐harm.

### 2.5 Statistical analysis

We first assessed the association between autism (overall, and with or without ID) and hospitalization for self‐harm using modified Poisson regression.^42^ We calculated relative risks (RRs) and used robust standard errors to derive 95% confidence intervals (CIs), taking account of clustering of individuals born to the same mother. Initial models were adjusted for age and sex (Model 1) followed by additional adjustment for birth order, maternal and paternal age, highest educational attainment of mother or father, disposable family income adjusted for family size at birth in quintiles, birth of parents or child outside of Sweden and a record of any psychiatric disorders in the mother or father (Model 2). We repeated these analyses for three subcategories: poisoning (ICD‐9: 950‐952; 980‐982 and ICD 10: X60‐X69; Y19‐Y19), cutting (ICD‐9: 956, 986; ICD‐10: X78; Y28) or violent forms of self‐harm (ICD‐9: 953‐957, 983‐987; ICD‐10: X70‐X75, X80, X81, Y20‐25, Y30, Y31).

In our second analysis, we investigated how three common psychiatric comorbidities‐ ADHD, depression, and anxiety disorders, influence the risk of self‐harm in autism. For each of these, we created variables with four categories: no autism or comorbidity, autism only, autism with the comorbidity, the comorbid condition without autism. We then estimated the associations of self‐harm with these variables using the approach described above.

In our third analysis, we assessed the risk of self‐harm in full and half siblings of individuals with autism as compared with the general population. Because full siblings share a greater proportion of their co‐segregating genes (50%), they are genetically more similar than half siblings who share 25% of their co‐segregating genes. Therefore, if the association between autism and self‐harm was primarily related to shared genetics, we would expect the relative risk of self‐harm in autism to be highest followed by non‐autistic full‐siblings and half‐siblings of people with autism. In the subset of families with more than one child, we matched each case of autism with up to 2 full same‐sex siblings who did not have a diagnosis of autism. We then carried out fixed effects conditional logistic regression analysis to directly compare the risk of self‐harm in individuals with autism with that of their non‐autistic full siblings by estimating odds ratios (ORs). For these analyses, we adjusted for age and birth order (model 1) and additionally for maternal and paternal age at birth (model 2) as these are likely to be non‐shared characteristics between sibling pairs. All analyses were conducted using Stata 14.^43^

## 3 RESULTS

For our main analyses, we had 410,732 individuals born to 245,117 mothers followed up in the registers from at least 10 to a maximum of 27 years of age. Of these, 9070 had a diagnosis of autism (6753 of whom had no ID and 2317 had a recorded diagnosis ID). Overall, individuals with autism were slightly younger, more likely to be male, come from families with lower education and parental history of a recorded psychiatric disorder than their peers. Individuals with autism and ID were more likely to come from families in the lowest quintile of family income and belong to migrant households than the general population, while the reverse was true for individuals with autism without ID (Table 1).

**TABLE 1:** Characteristics of the eligible study population within the Stockholm Youth Cohort, by autism with or without intellectual disability (ID)

	No Autism	All Autism	Autism without ID	Autism with ID
	(n = 401,662)	(n = 9,070)	(n = 6,753)	(n = 2,317)
*Characteristic*				
Age at f/u (mean, *SD*)	18.2 (5.0)	17.2 (4.6)	17.0 (4.6)	17.6 (4.6)
Male sex	50.80	69.14	68.30	71.60
First born	44.86	44.71	43.45	48.38
Mother's age at birth (mean, *SD*)	29.2 (5.2)	29.5 (5.5)	29.4 (5.4)	29.7 (5.6)
Father's age at birth (mean, *SD*)	32.2 (6.3)	32.5 (6.7)	32.3 (6.6)	33.3 (7.0)
Low education parents	29.54	31.75	30.73	34.74
Lowest quintile of family income	20.79	17.55	15.49	23.56
Individual or their parents born outside Sweden	36.92	33.14	29.08	44.97
Mother has psychiatric history	33.55	50.07	52.32	43.50
Father has psychiatric history	21.74	30.86	31.48	29.05
*Admission for self‐harm during follow‐up (%)*				
Any admission for self‐harm^a^	0.76	2.97	3.55	1.25
Known intent	0.67	2.80	3.35	1.21
Unknown intent	0.12	0.33	0.39	0.17
Self‐harm by cutting	0.06	0.45	0.52	0.26
Poisoning	0.67	2.59	3.12	1.04
Violent self‐harm	0.03	0.20	0.24	0.09

*Note*: (1) All numbers are column percentages, except age variables which refer to the mean (standard deviation).

^a^ Refers to at least one admission for any type of self‐harm regardless of intent. Calculated by date of first episode and therefore proportions below will not add up.

Nearly 3% of autistic individuals had been hospitalized at least once for self‐harm between the ages of 10 and 27 years, compared with 0.8% of their non‐autistic peers (Table 1). Hospitalizations for self‐harm were more common in autistic individuals without ID (3.6%) than in those who had both diagnoses (1.3%). Although, poisoning was the most common self‐harm method among admitted individuals regardless of autism, over 15% of all admissions in the autistic group were because of self‐cutting compared with 8% in the non‐autistic group.

Table 2 shows the RRs for admission because of self‐harm in autistic individuals (autism without or with ID), as compared with the non‐autistic population. Individuals with autism had an almost a fivefold adjusted relative risk for admissions (RR 5.0 [95% CI 4.4–5.6]) which was greatest for autism without ID (5.9 [5.2–6.7]), but also increased for autism with ID (RR 1.9 [1.3–2.7]). A pattern of high RR was evident in analyses for all the methods of self‐harm we examined (poisoning, cutting and violent forms of self‐harm), however, RR related to self‐cutting and violent methods of self‐harm were a particularly high in the autistic compared with the non‐autistic population (Table 2).

**TABLE 2:** Relative risks (RRs) of self‐harm in relation to autism (with and without intellectual disability [ID]), by method of self‐harm

	All autism		Autism without ID		Autism with ID	
	Model 1^a^	Model 2^b^	Model 1	Model 2	Model 1	Model 2
	RR(95%CI)^c^	RR (95% CI)	RR(95% CI)	RR(95% CI)	RR(95% CI)	RR (95% CI)
All self‐harm^d^	5.88 (5.20–6.64)	4.95 (4.37–5.61)	7.08 (6.23–8.04)	5.91 (5.19–6.74)	2.22 (1.55–3.19)	1.93 (1.34–2.77)
Self‐harm by cutting^e^	11.74 (8.39–16.41)	9.35 (6.66–13.12)	13.25 (9.27–18.94)	10.24 (7.12–14.72)	5.63 (2.51–12.63)	4.86 (2.17–10.88)
Self‐harm by poisoning^f^	5.96 (5.23–6.80)	5.00 (4.38–5.72)	7.22 (6.30–8.27)	6.01 (5.22–6.91)	2.15 (1.44–3.20)	1.85 (1.24–2.76)
Violent self‐harm^g^	8.56 (5.20–14.08)	7.49 (4.51–12.44)	10.29 (6.10–17.35)	8.99 (5.24–15.37)	3.10 (0.76–12.57)	2.72 (0.67–11.00)

^a^ Model 1 = age and sex adjusted model.

^b^ Model 2 = adjusted for age, sex, maternal and paternal age, parental educational attainment around birth, quintile of disposable household income around birth, own or parental foreign birth, and maternal and paternal history of psychiatric care.

^c^ Relative risk with robust 95% confidence interval.

^d^ Includes: ICD‐9950–958 & 980–988 and ICD‐10 X60‐X84 & Y10‐Y34.

^e^ Includes: ICD‐9956 & 986 and ICD‐10 X78 & Y28.

^f^ Includes: ICD‐9950–952 & 980–982 and ICD‐10 X60‐X69 & Y10‐Y19.

^g^ Includes: ICD‐9953–957 & 983–987 and ICD‐10 X70‐X75, X80, X81, Y20‐Y25, Y30 & Y31.

Risks of self‐harm in autism (with and without ID) cross‐classified according to psychiatric comorbidities are shown in Table 3. The risk of self‐harm was increased in autism, particularly in the subgroup without ID, also when occurring without other comorbidities. Risks were about fivefold and similar for single diagnoses of either autism without ID (RR 5.9 [95% CI 4.9–7.0]), or ADHD (6.1 [5.6–6.8]), or depression (4.6 [4.2–5.0]), and somewhat lower in anxiety disorders (3.0 [2.8–3.3]). Individuals with autism without ID and either ADHD, depression, or anxiety disorder additionally, had about a tenfold increase in risk for self‐harm as compared with those without any diagnoses in the fully adjusted model (Model 2).

**TABLE 3:** Relative risks (RRs) of self‐harm in relation to autism, with and without intellectual disability [ID] and other psychiatric disorders

	All autism		Autism without ID		Autism with ID	
	Model 1^a^	Model 2^b^	Model 1^a^	Model 2^b^	Model 1^a^	Model 2^b^
	RR (95% CI)^c^	RR (95% CI)^c^	RR (95% CI)^c^	RR (95% CI)^c^	RR (95% CI)^c^	RR (95% CI)^c^
*ADHD*						
No autism or ADHD	1.00	1.00	1.00	1.00	1.00	1.00
Autism without ADHD	5.15 (4.34–6.11)	4.61 (3.88–5.47)	6.53 (5.47–7.81)	5.85 (4.86–6.96)	1.74 (1.04–2.93)	1.58 (0.94–2.66)
ADHD without autism	8.09 (7.35–8.91)	6.21 (5.62–6.86)	7.97 (7.25–8.77)	6.14 (5.56–6.77)	8.27 (7.58–9.02)	6.35 (5.80–6.96)
Autism with ADHD	10.51 (8.90–12.41)	8.34 (7.04–9.89)	11.64 (9.79–13.85)	9.25 (7.74–11.05)	5.57 (3.36–9.23)	4.43 (2.68–7.32)
*Depression*						
No autism or depression	1.00	1.00	1.00	1.00	1.00	1.00
Autism without depression	5.54 (4.70–6.50)	4.79 (4.06–5.65)	6.99 (5.89–8.30)	5.99 (5.04–7.13)	2.09 (1.33–3.28)	1.85 (1.18–2.89)
Depression without autism	5.40 (4.97–5.86)	4.57 (4.18–4.96)	5.40 (4.97–5.86)	4.56 (4.19–4.96)	5.56 (5.14–6.02)	4.67 (4.30–5.07)
Autism with depression	13.25 (11.10–15.80)	11.19 (9.33–13.41)	13.67 (11.38–16.43)	11.59 (9.60–14.00)	9.10 (5.03–16.44)	7.51 (4.10–13.74)
*Anxiety Disorders*						
No autism or anxiety	1.00	1.00	1.00	1.00	1.00	1.00
Autism without anxiety	5.80 (4.95–6.78)	4.96 (4.24–5.82)	7.31 (6.20–8.62)	6.17 (5.22–7.29)	2.14 (1.38–3.31)	1.90 (1.23–2.93)
Anxiety without autism	3.55 (3.27–3.85)	2.99 (2.75–3.26)	3.55 (3.27–3.85)	3.00 (2.75–3.26)	3.67 (3.39–3.97)	3.07 (2.84–3.34)
Autism with anxiety	10.74 (8.89–12.97)	8.90 (7.33–10.79)	11.33 (9.30–13.80)	9.51 (7.78–11.63)	6.33 (3.37–11.87)	4.86 (2.57–9.21)

*Notes*: Hospital admission for self‐harm (ICD‐9950–958 & 980–988 and ICD‐10 X60‐X84 & Y10‐Y34).

^a^ Model 1 = age and sex adjusted model.

^b^ Model 2 = adjusted for age, sex, maternal and paternal age, parental educational attainment around birth, quintile of disposable household income around birth, own or parental foreign birth, and maternal and paternal history of psychiatric care.

^c^ 95% confidence interval.

Risks for self‐harm in the full and half siblings of individuals with autism, who themselves had never received an autism diagnosis, are presented in Table 4. Non‐autistic full (1.6 [1.3–2.0]) and half siblings (1.4 [1.1–1.9]) of individuals with autism had a higher risk of self‐harm as compared with population controls. These associations were more apparent for siblings of children with autism without intellectual disability (Table 4).

**TABLE 4:** Relative risks (RRs) of self‐harm in relation to autism with and without intellectual disability [ID] and having a sibling with autism

	All autism		Autism without ID		Autism with ID	
	Model 1^a^	Model 2^b^	Model 1^a^	Model 2^b^	Model 1^a^	Model 2^b^
	RR (95% CI)^c^	RR (95% CI)^c^	RR (95% CI)^c^	RR (95% CI)^c^	RR (95% CI)^c^	RR (95% CI)^c^
Autistic individuals	5.88 (5.20–6.64)	4.95 (4.37–5.61)	7.08 (6.23–8.04)	5.91 (5.19–6.73)	2.22 (1.55–3.19)	1.93 (1.34–2.77)
Siblings of autistic individuals	1.76 (1.43–2.18)	1.57 (1.27–1.95)	1.95 (1.53–2.48)	1.70 (1.33–2.16)	1.30 (0.84–2.00)	1.22 (0.79–1.88)
Half‐siblings of autistic individuals	2.00 (1.50–2.67)	1.40 (1.05–1.87)	2.10 (1.54–2.87)	1.45 (1.06–1.98)	1.62 (0.83–3.19)	1.22 (0.62–2.39)

*Notes*: Hospital admission for self‐harm: ICD‐9950–958 & 980–988 and ICD‐10 X60‐X84 & Y10‐Y34.

^a^ Model 1 = age and sex adjusted model.

^b^ Model 2 = adjusted for age, sex, maternal and paternal age, parental educational attainment around birth, quintile of disposable household income around birth, own or parental foreign birth, and maternal and paternal history of psychiatric care.

^c^ Relative risk with robust 95% confidence interval.

Finally, we directly compared the risk of self‐harm in autistic individuals (*n* = 2902) to that in matched same‐sexed full siblings without a diagnosis of autism (*n* = 3410). Autistic individuals had an over a twofold risk of self‐harm (adjusted OR 2.2 [1.5–3.3]) compared with their non‐autistic siblings (Table S1).

## 4 DISCUSSION

This large population‐based study demonstrates a clear overrepresentation of hospital admissions and emergency department visits for self‐harm in adolescents and young adults with autism in comparison to their typically developing peers. This increased risk was evident in autistic individuals without any recorded diagnosis of ADHD, depression, or anxiety disorders, which are well established as risk‐factors for self‐harm. The magnitude of single risks of self‐harm in autism were equipollent the risks that were observed in ADHD, depression, and anxiety disorders per se, indicating that autism might be an independent risk factor for self‐harm. The risk of self‐harm was particularly high in autistic individuals without ID although, an increased risk was observed also in individuals with autism with ID, compared with non‐autistic individuals. Furthermore, sibling‐analyses indicated that the increased risk of self‐harm in autism largely reflects factors specific to these conditions rather than shared familial genetic or environmental factors. Lastly, although the most common method of self‐harm was self‐poisoning in autistic and non‐autistic individuals alike, relative risks of self‐harm by especially cutting but also by violent methods (gassing, hanging, strangulation/suffocation, drowning, jumping and firearms) were particularly increased in the autistic group.

Our overall results, including a particularly marked risk increase of self‐harm among autistic individuals without ID, confirm those of a previous case–control study from Sweden.^27^ Yet, our study extends its findings by focusing on a recent population diagnosed according to today's more inclusive diagnostic practices. We furthermore base our analyses on a more detailed case ascertainment, also including data from outpatient health services (comprising child and adolescent as well as adult psychiatric care) as opposed to prior work that has mainly relied on diagnoses retrieved solely from inpatient care. Lastly, we adjusted estimates of associations for a fuller set of sociodemographic factors such as parental educational attainment and disposable family income adjusted for family size.

We found that autistic individuals were more likely to be admitted to hospitals/emergency departments for self‐harm regardless of common comorbid psychiatric disorders, which is consistent with recent research from Taiwan.^23^ Our results, however, contrast those of a smaller Finnish study demonstrating that the increased risk of intentional self‐harm was attenuated with adjustment for psychiatric comorbidities.^22^ The Finnish study's approach of adjusting for comorbidities that can be regarded as mediators of the relationship between autism and self‐harm may have introduced collider bias that explain discrepancies between their and our findings. In our study, an attempt to introduce temporal alignment of recorded events (the diagnosis of comorbid disorder such as depression preceded the record of self‐harm event), contrasting other register‐based studies^22^, ^23^, ^27^ (adjusting for life‐time comorbidity). Our approach aimed to minimize any possibility of reverse causality leading to spurious results. However, we reported equally concerning risk increase for self‐harm.

Our comparisons of autistic individuals with their siblings and half‐siblings indicate that autism is associated to self‐harm independently of shared familial factors (i.e., the 50% of genes and the aspects of the family environment, such as parenting style or living conditions, that is shared by siblings).

Yet, we also show, in line with others,^27^ that such factors have some impact as siblings of autistic individuals were somewhat more likely to have self‐harmed than the general population.^27^ As we did not observe any clear gradient between full and half‐siblings, shared environmental factors, rather than genes, may be important for self‐harm in autism.

Self‐harm methods used by autistic individuals have not been well characterized up to date.^44^ Self‐cutting and poisoning are the most prevalent methods, in community and hospital‐based studies, respectively.^4^, ^45^ In accordance, poisoning was by far the most common self‐harm method in our study of hospital admissions. Yet, autistic individuals in our study, and especially those without ID, had a more notable increased risk for admissions because of self‐cutting than poising. This subgroup seemingly also had a very high risk of admissions because of violent self‐harm, although this finding was based on small numbers. The results are noteworthy since violent self‐harm has a poorer prognosis,^28^, ^46^ and since self‐cutting may be more predictive of suicide in autism than among typically developing people.^44^

### 4.1 Strengths and limitations

A major strength of this study is the population‐based design and inclusion of a large contemporary sample where autism was comprehensively ascertained. Self‐harm was prospectively recorded by clinicians and retrieved from a nationwide health care register, minimizing any recall or reporting bias. We were also able to reduce bias from confounding, since we had access to and adjusted for a range of individual and family level variables and could use siblings as a comparison group to adjust for familial confounding. Methodological concerns, other than poor control for psychiatric comorbidities and other confounding factors, such as small and selected samples and cross‐sectional design also hamper interpretation of results from many prior studies.^17^, ^20^, ^30^

However, the results need to be interpreted in the light of some limitations. Since we could only include incidents of self‐harm which present to emergency departments or otherwise result in in‐patient care, the prevalence of self‐harm in our study is almost certainly an under‐estimate. This outcome misclassification may be nondifferential, that is, if the rate of underreporting may be similar in individuals with and without autism, and, if so, result in an underestimation of any true association between autism and self‐harm. It may, however, also be that autistic people are more or less likely than their peers to seek emergency and/or receive in‐patient care for self‐harm issues. If, for example, individuals with autism are more likely than the general population to be admitted for self‐harm, for example, because of a more extensive surveillance by health‐care services, our results may instead overestimate true associations.

Misclassification may concern identification of comorbidities in this register‐based study, as also mental disorders other than self‐harm may be differentially detected by services. Furthermore, stratification or adjustment for a mediator may introduce collider stratification bias in any study, this requires that also our findings as those of other researchers, regarding the relative risks of self‐harm in presence or absence of comorbid conditions should be interpreted with caution.

In conclusion, in this total population‐based study of young individuals we found that autism was associated to increased risk of self‐harm independently of comorbid ADHD, depression and anxiety disorders and that methods of self‐harm used by autistic individuals, particularly those without intellectual disability, may be more lethal that those used by non‐autistic peers. Together, these findings indicate that self‐harm, an important predictor of suicide in the general population, if present in young autistic individuals should be a matter of great clinical concern as well as prompt and adequate safe‐planning actions.

## AUTHOR CONTRIBUTIONS

Isidora Stark, Dheeraj Rai, Selma Idring Nordström and Cecilia Magnusson designed the study. Michael Lundberg, Isidora Stark, Dheeraj Rai, and Cecilia Magnusson had full access to the data and were responsible for data integrity and statistical analyses. Isidora Stark, Dheeraj Rai, Selma Idring Nordström, Iryna Culpin, Michael Lundberg, Anna Ohlis and Cecilia Magnusson, interpreted the data. Isidora Stark wrote the manuscript and integrated feedback from the other authors. All authors were involved in critical revision of the study design and manuscript drafts and approved the final version.

### PEER REVIEW

The peer review history for this article is available at https://publons.com/publon/10.1111/acps.13479.

## Supporting information


**Table S1** Odds ratios (OR) of hospital admissions for self‐harm among autistic cases compared with sex‐matched full sibling controls

## Data Availability

The data that support the findings of this study are available on request from the corresponding author. The data are not publicly available due to privacy or ethical restrictions.
