# Supplementary material for: Autism and self‐harm: A population‐based and discordant sibling study of young individuals
Source: Acta Psychiatr Scand. 2022 Aug 3;146(5):468–77. doi: 10.1111/acps.13479 (PMC10286753; doi:10.1111/acps.13479)
Supplement: Supplementary file 1 — Table S1 Odds ratios (OR) of hospital admissions for self‐harm among autistic cases compared with sex‐matched full sibling controls [file ACPS-146-468-s001.docx]

Supplementary table 1:

Odds ratios (OR) of hospital admissions for self-harm among autistic cases compared with sex-matched full sibling controls

|  | OR (95% CI)  **Model 1** | OR (95% CI)  **Model 2** | n / N  (autistic cases) | n / N  (sibling controls) |
| --- | --- | --- | --- | --- |
| Autism | 2.18 (1.47-3.25) | 2.18 (1.46-3.25) | 80/2,902 | 49/3,410 |
| Autism without ID | 2.94 (1.82-4.74) | 2.94 (1.82-4.75) | 71/1,951 | 33/2,289 |
| Autism with ID | 0.78 (0.30-1.99) | 0.70 (0.27-1.81) | 7/852 | 12/996 |

Notes: (1) Conditional logistic regression. (2) **Model 1=** adjusted for age and birth order. (3) **Model 2=** adjusted for age, birth order, maternal and paternal age. (4) n= number of hospital admissions for self-harm ((ICD-9 950-958 & 980-988 and ICD-10 X60-X84 & Y10-Y34). N= total number at risk.
